# Multi-omics analysis of a fatty liver model using human hepatocyte chimeric mice

**DOI:** 10.1038/s41598-024-53890-8

**Published:** 2024-02-09

**Authors:** Akemi Ichikawa, Daiki Miki, C. Nelson Hayes, Yuji Teraoka, Hikaru Nakahara, Chise Tateno, Yuji Ishida, Kazuaki Chayama, Shiro Oka

**Affiliations:** 1 Department of Gastroenterology, Graduate School of Biomedical and Health Science, 1-2-3 Kasumi, Minami-ku, Hiroshima, 734-8551 Japan; 2 Pfizer, Inc., Tokyo, Japan; 3 Department of Clinical and Molecular Genetics, Hiroshima University, Hiroshima, Japan; 4 PhoenixBio Co., Ltd., Higashihiroshima, Japan; 5 Collaborative Research Laboratory of Medical Innovation, Hiroshima University, Hiroshima, Japan; 6 RIKEN Center for Integrative Medical Sciences, Yokohama, Japan

**Keywords:** Non-alcoholic fatty liver disease, Animal disease models

## Abstract

We developed a fatty liver mouse model using human hepatocyte chimeric mice. As transplanted human hepatocytes do not respond to mouse growth hormone (GH) and tend to accumulate fat, we hypothesized that addition of human GH would alter lipid metabolism and reduce accumulation of fat in the liver even when fed a high-fat diet. Six uPA/SCID chimeric mice were fed a high-fat GAN diet to induce fatty liver while six were fed a normal CRF1 diet, and GH was administered to three mice in each group. The mice were euthanized at 8 weeks, and human hepatocytes were extracted for RNA-Seq, DIA proteomics, and metabolomics analysis. Abdominal echocardiography revealed that the degree of fatty liver increased significantly in mice fed GAN diet (*p* < 0.001) and decreased significantly in mice treated with GH (*p* = 0.026). Weighted gene correlation network analysis identified *IGF1* and *SEMA7A* as eigengenes. Administration of GH significantly reduced triglyceride levels and was strongly associated with metabolism of amino acids. MiBiOmics analysis identified perilipin-2 as a co-inertia driver. Results from multi-omics analysis revealed distinct gene expression and protein/metabolite profiles in each treatment group when mice were fed a high-fat or normal diet with or without administration of GH.

## Introduction

Nonalcoholic fatty liver disease (NAFLD) is an inflammatory condition that can progress to cirrhosis and hepatocellular carcinoma and is a leading indication for liver transplantation^1^. Concomitant with the rise in obesity, the global incidence of NAFLD is rapidly increasing and now affects up to one-quarter of the world’s population^2,3^. NAFLD, defined as the build-up of fat in the liver in the absence of excess alcohol, is the hepatic manifestation of metabolic syndrome and is often associated with obesity but is also observed in a subset of patients without obesity^4^.

In 2020, an international team proposed redefining this condition as metabolic-associated fatty liver disease (MAFLD) to emphasize the central role of metabolic dysregulation^5,6^. However, the terms NAFLD and MAFLD are not strictly interchangeable, as the diagnostic criteria for MAFLD also require being overweight/obese, having type 2 diabetes mellitus, or having two out of seven specific metabolic abnormalities^7^. Importantly, however, unlike NAFLD, MAFLD does not preclude concomitant liver diseases due to alcohol, hepatitis B virus (HBV), hepatitis C virus (HCV), or autoimmune hepatitis^7^. Therefore, a larger proportion of patients qualify for a diagnosis of MAFLD than for NAFLD^7^. Although controversial, this more inclusive definition more directly reflects the underlying state of atherogenic dyslipidemia and insulin resistance, which is also associated with increased risk of cardiovascular disease and cancer^8^. Therefore, investigating hepatic mechanisms regulating lipid metabolism may yield advances in preventing and treating extrahepatic manifestations through lifestyle changes and targeted therapies.

The increasing incidence of obesity and MAFLD suggest the need for new treatments to manage MAFLD and its complications, but few options are currently available^9^. One reason for this is the complex and incompletely understood pathogenesis of the disease and the involvement of multiple tissues.

Small animal models are essential for the development and evaluation of new treatments for NAFLD/MAFLD. A range of small animal NAFLD models have been developed, including a diet model, a genetic model, a genetic and dietary model, and a chemical-based model^10,11^. The high-fat, high-cholesterol (HFHC) diet model has been developed as a model for high-fat diet-induced non-alcoholic steatohepatitis (NASH) and used for analysis of insulin resistance associated with obesity^12^. Genetically modified mouse models have also been developed to study NASH/NAFLD^12–14^. Although these models are valuable tools to study NAFLD/MAFLD, metabolism in mice and humans differs in fundamental ways. We have previously established a chimeric mouse model in which the liver of a DNA-urokinase-type plasminogen activator (uPA)/severe combined immunodeficiency (SCID) mouse is repopulated with human hepatocytes^15,16^, which we have successfully used to investigate HBV and HCV infection in vivo and to evaluate antiviral agents^17,18^.

Our goal in this study was to adjust this experimental system to develop a fatty liver mouse model that more closely approximates the pathophysiology of MAFLD in humans. Human hepatocyte chimeric mice already exhibit mild to moderate fatty liver, as the human GH receptor does not respond to mouse GH, resulting in symptoms of GH deficiency^19^. Matsumoto et al. reported that human IGF-1 was not detectable in the sera of uPA/SCID chimeric mice^20^ and that injection of recombinant human GRP increased the repopulation rate and up-regulated expression of human IGF-1, STAT1, STAT3, and several cell cycle regulatory genes. Tateno et al.^19^ also reported that transplanted hepatocytes entered a GH-deficient state leading to steatosis, while administration of GH drastically reduced the accumulation of fat. We take advantage of this as the basis of our model, allowing us to examine the effect of human GH in isolation as well as to examine the role of GH in helping the liver to process a high-fat diet. We divided mice into four groups according to whether or not they received GH and GAN diet and investigated the metabolic state within each group using a multi-omics approach.

## Results

All mice survived until the end of the experiment. GAN-fed mice showed rapid initial weight gain while CRF1-fed mice gained weight more gradually (Suppl. Fig. 1). Human serum albumin levels remained relatively constant throughout the experiment, although the level in one mouse in the GH + /GAN-fed group decreased slightly (Suppl. Fig. 2).

### Abdominal ultrasound results

**Figure 1:**
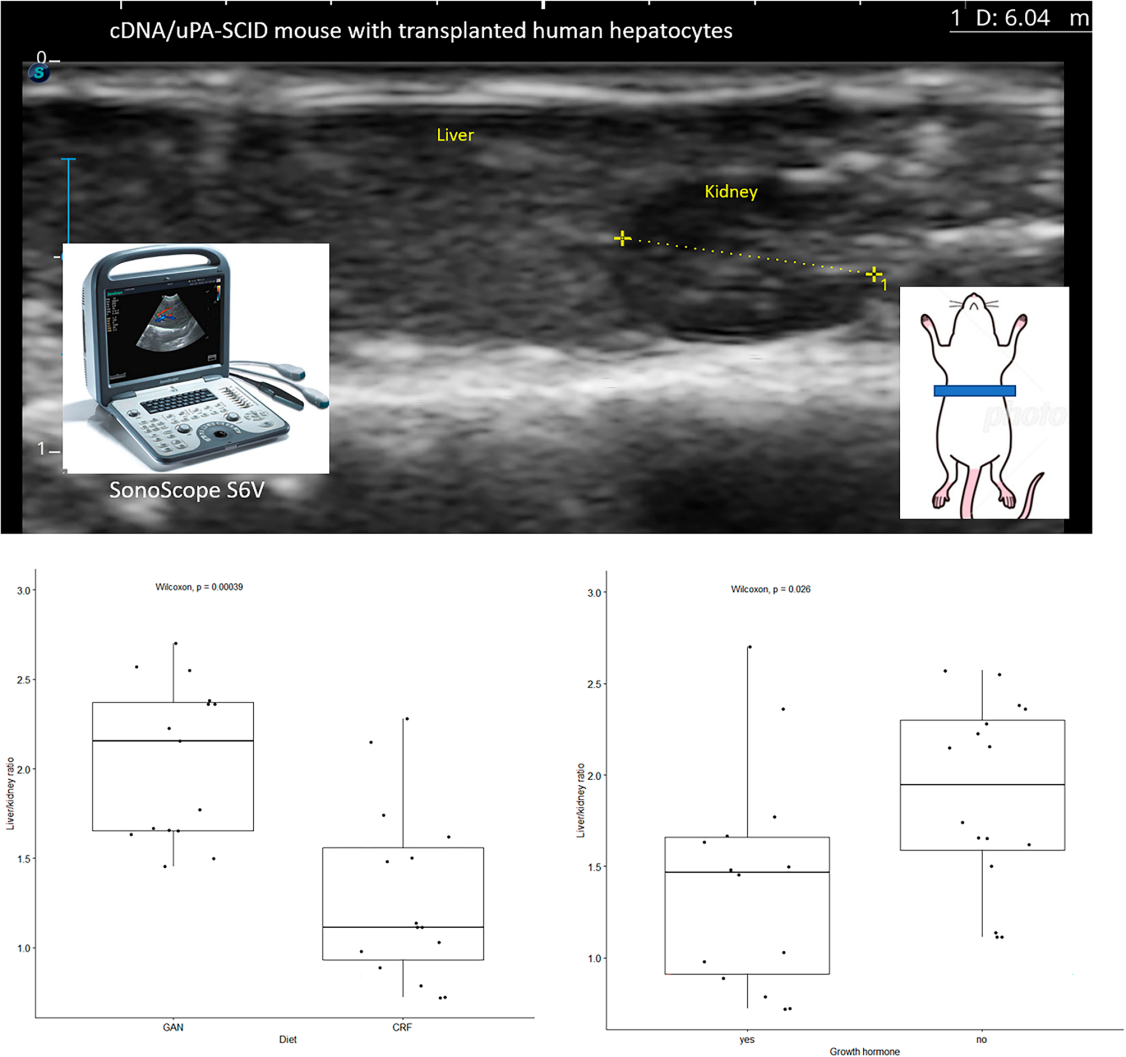
Estimation of the degree of fatty liver in a chimeric mouse model. (**A**) The degree of fatty liver was estimated by calculating the liver to kidney ratio in ultrasound images. (**B**) Ultrasound liver/kidney ratio with respect to GAN versus CRF1 diet. (**C**) Ultrasound liver/kidney ratio with respect to growth hormone administration.

The degree of fatty liver was estimated in vivo using abdominal echocardiography (Fig. 1A). Collectively, the liver/kidney ratio was significantly greater in GAN-fed mice (*p* < 0.001) (Fig. 1B) and in mice that were not treated with GH (*p* = 0.026) (Fig. 1C). In pairwise analysis, administration of GH significantly reduced fatty liver in CRF1-fed mice (*p* = 0.022), but there was no significant difference in GAN-fed mice (*p* = 0.42) (Suppl. Fig. 3).

### Histological findings

**Figure 2:**
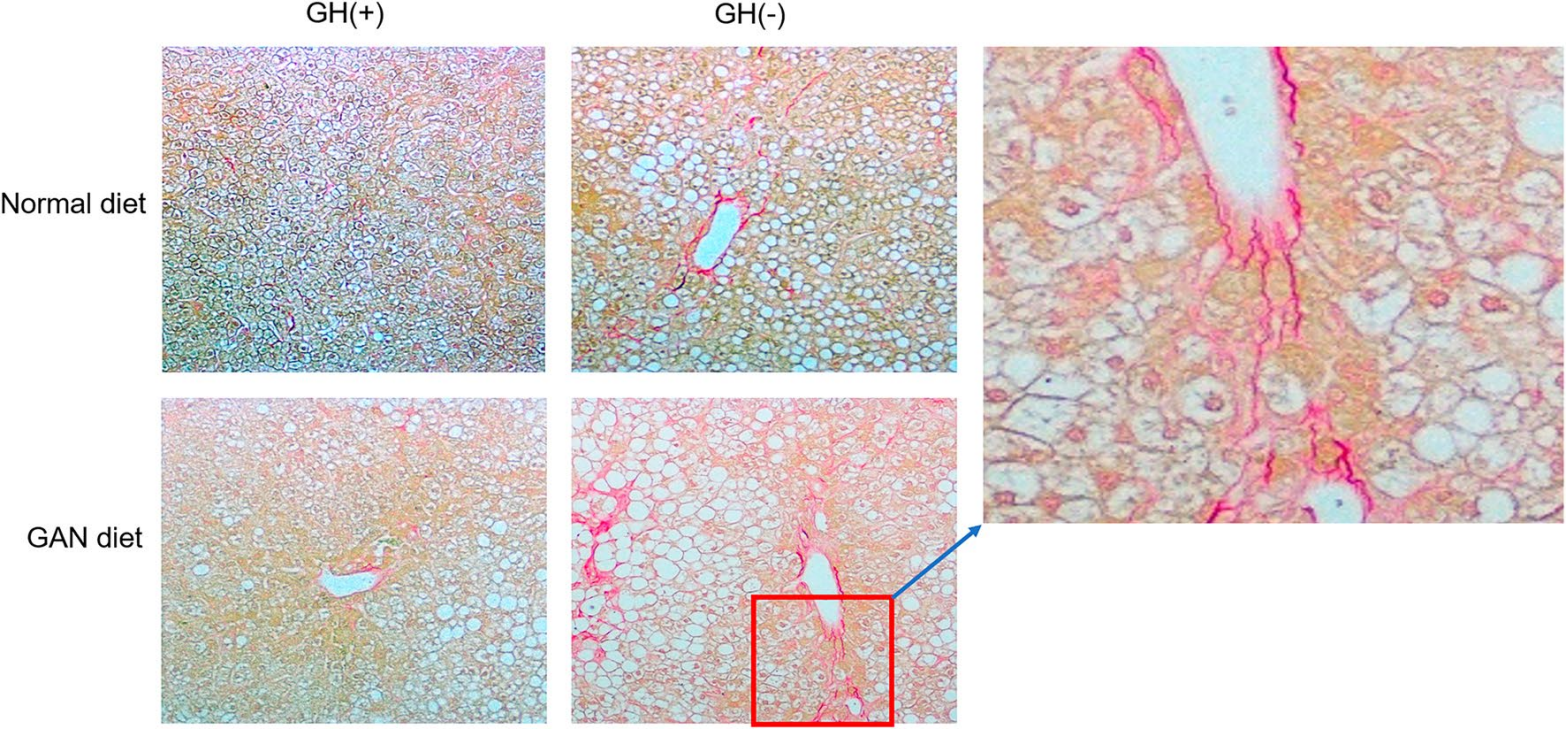
Histology of fatty liver in a chimeric mouse model. Humanized liver tissue was extracted at 8 weeks and stained with Sirius-Red staining with respect to GH ±  and CRF1/GAN diet. The inset shows collagen staining around the venule.

Optical microscopy of liver tissue showed differences in cell morphology among treatment groups. Macrovesicular steatosis in perivenular hepatocytes with large fat droplets and displaced nuclei was observed in mice fed GAN diet as well as in GH− mice fed a normal CRF1diet (Fig. 2). The dark magenta strands indicate densely staining fibrotic connective tissue surrounding the venule in GH− mice. Sirius Red staining was more extensive in GH−/GAN-fed mice, radiating away from the venule and revealing a region of pericellular “chicken-wire” fibrosis surrounding large hepatocytes.

### Electron microscopy results

**Figure 3:**
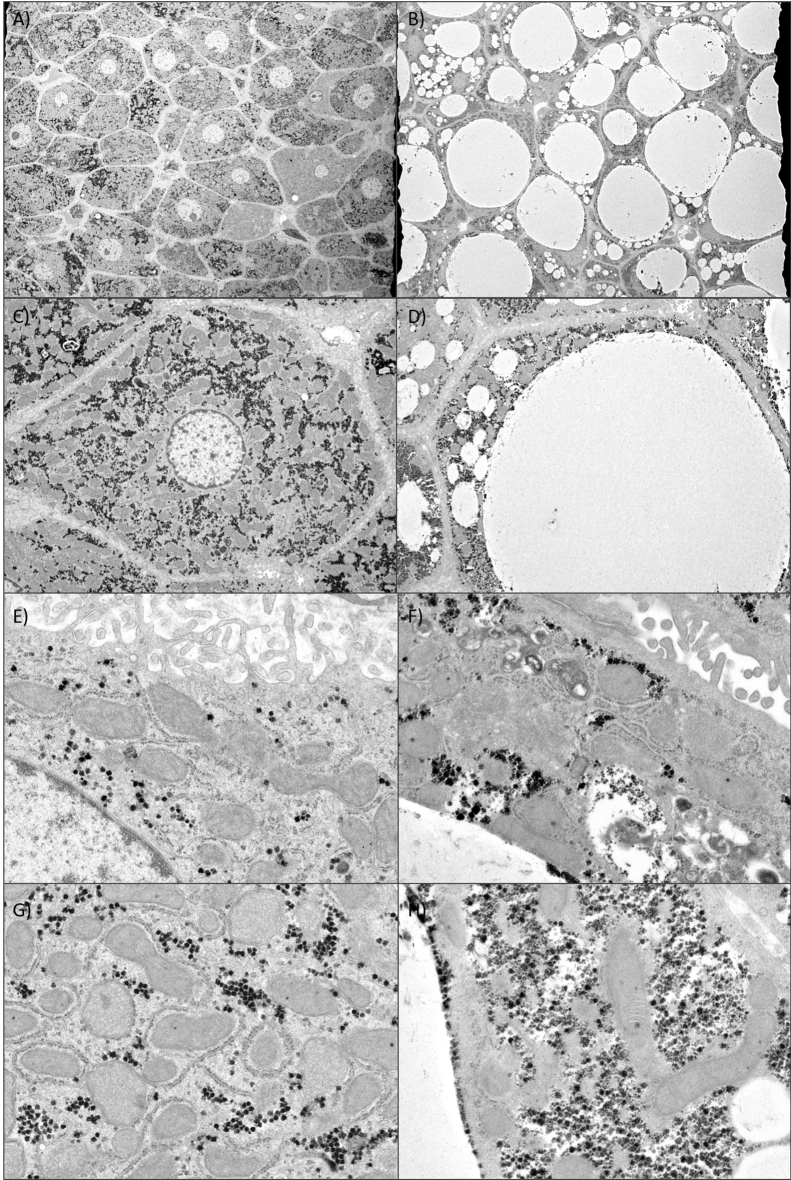
Electron microscopy of fatty liver in a chimeric mouse model. Electron micrographs of human hepatocyte-transplanted mouse liver tissue extracted from a GH+/CRF1-fed mouse (left column) and a GH−/GAN-fed mouse (right column). (**A**) Liver section showing normal hepatocytes. (**B**) Liver section showing hepatocytes with large fat droplets. (**C**–**H**) Enlarged views of individual hepatocytes.

Electron microscopy of liver tissue from a GH−/GAN-fed mouse and a GH+/CRF1-fed mouse revealed dramatic differences in cell morphology (Fig. 3). One or more large fat droplets with displaced nuclei were observed in the GH−/GAN mouse, and hepatocytes were larger and more irregularly shaped. The pericellular space was denser, with fewer and more stunted microvilli. Mitochondria tended to be smaller and less abundant in GH−/GAN mice but were characterized by a small number of unusually large mitochondria, often with pronounced internal features, including what appears to be a large crystalline inclusion body. Rough endoplasmic reticulum was clearly visible adjacent to mitochondria in GH+/CRF1 mice but was less discernable in GH−/GAN mice.

### Transcriptomics (RNA-seq)

RNA sequencing generated about 40 million reads/sample. About 60% of reads mapped to the hg19 human reference sequence. Changes in gene expression were examined with respect to GAN or CRF1 diet and administration of GH (Suppl. Table 1). The most notable differences were observed between GH−/GAN-fed (severe fatty liver) mice and GH+/CRF1-fed (no fatty liver) mice, resulting in differential gene expression in 950 genes, many of which are involved in glucose and lipid metabolism. The next largest difference was with respect to GAN diet versus CRF1 diet in GH+ mice, with 269 up- or -down-regulated genes involved mainly in lipid-related functions. Finally, in mice fed CRF1 diet, treatment with GH resulted in differential expression of 121 genes compared to GH− mice.

### Weighted gene co-expression network analysis results

**Figure 4:**
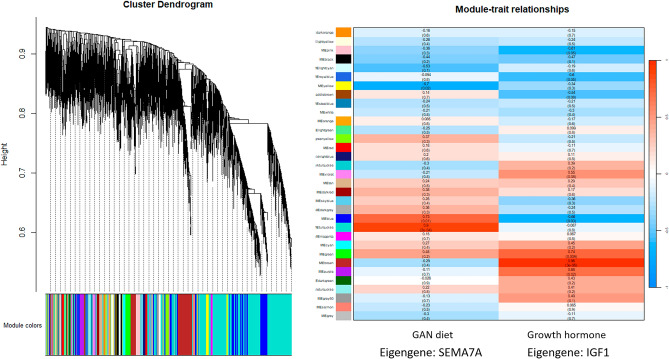
Weighted gene co-expression network analysis of transcriptomics data. (**A**) WGCNA cluster dendrogram showing classification into 33 modules. (**B**) WGCNA module-trait relationships showing association between GAN diet and the MEturquoise module (eigengene: SEMA7A) and between growth hormone administration and the MEbrown module (eigengene: IGF1).

Weighted gene co-expression network analysis was performed using WGCNA version 1.70-3^21,22^. The gene expression network was transformed using a soft-thresholding power estimate of 3, resulting in classification into 33 modules (Fig. 4A). One mouse in the GH+/GAN-fed group was excluded from WGCNA analysis based on branch height using the cutreeStatic method according to the instructions. IGF1 and SEMA7A were identified as the eigengenes of modules associated with GH and GAN diet, respectively (Fig. 4B).

### Simple DIA protein analysis

Proteomics results were generally consistent with results from transcriptomics analysis (Suppl. Table 2). NanoLC-MS analysis by Scaffold DIA identified 3747 human proteins with FDR < 1%. In pairwise comparisons, 385 proteins differed with respect to GAN diet versus CRF1 diet in GH + mice, whereas 217 proteins differed in GH+ versus GH− mice CRF1-fed mice. The most extreme difference was observed between GH−/GAN diet versus GH+/CRF1, which showed differences in the levels of 878 proteins.

### Metabolomics

Metabolomics results were assembled based on a database of 1300 standard metabolites, of which 437 were detected in this study. Compared to the changes in gene expression, changes in metabolites varied considerably with respect to diet and GH. Administration of GH significantly reduced triglyceride levels and was strongly associated with metabolism of amino acids, particularly D-arginine and D-ornithine.

The most pronounced differences were between the GH + versus GH− mice in the GAN diet group and between GAN versus CRF1 diet mice in the GH− group (Suppl. Table 3). Conversely, in the GH + groups, the differences were primarily characterized by a strong increase in phosphatidylcholine (40:6), and the addition of GH was associated with a marked increase in 1-Methylnicotinamide in CRF1-fed mice.

### MetaboAnalyst results

**Figure 5:**
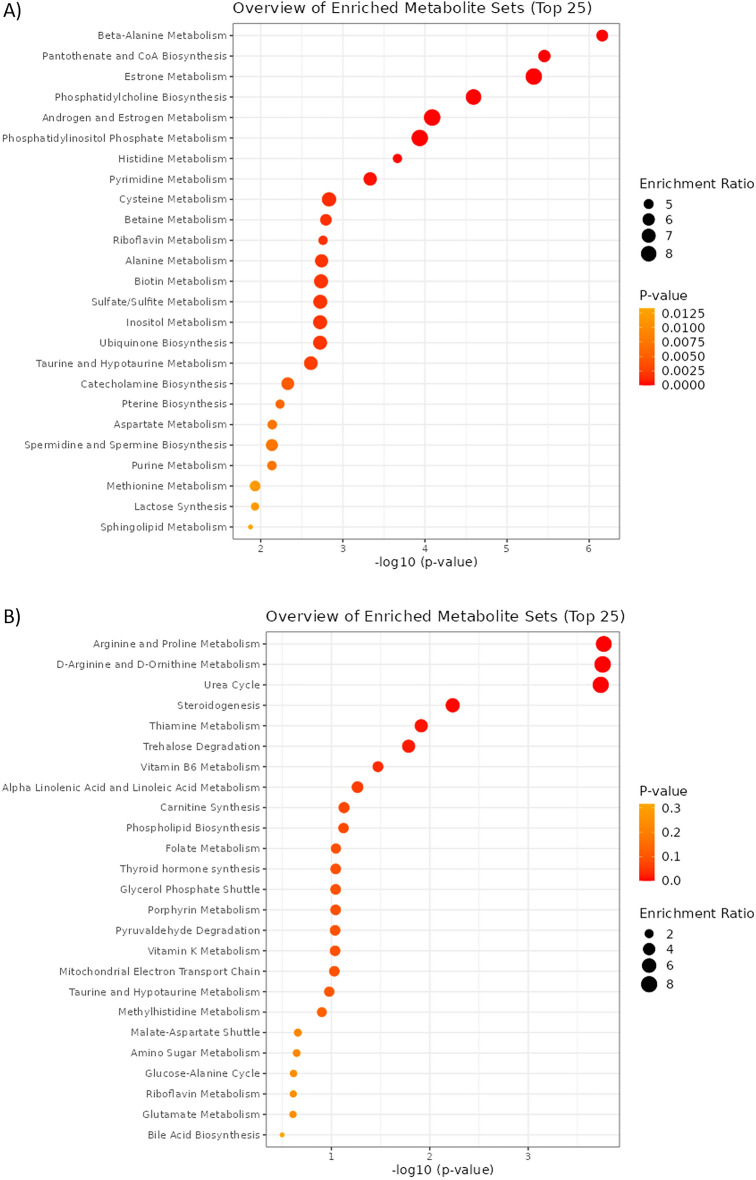
MetaboAnalyst quantitative enrichment of metabolite sets. Enriched metabolite sets associated with (**A**) GAN diet versus CRF1 diet, and (**B**) with or without administration of GH.

MetaboAnalyst version 5 was used to analyze differences in metabolites with respect to diet and GH administration^23^. Quantitative enrichment analysis revealed that beta-alanine metabolism, pantothenate and CoA biosynthesis, and estrone metabolism metabolite sets were enriched in GAN versus CRF1 diet (Fig. 5A), whereas arginine and proline metabolism, D-arginine and D-ornithine metabolism, and urea cycle metabolite sets were enriched in GH + versus GH− mice (Fig. 5B). The Statistical Analysis with Metadata module was used to identify individual metabolites associated with diet and GH. Pantothenate was found to be associated with diet (Suppl. Fig. 4A), while phosphatidylethanolamine was associated with GH (Suppl. Fig. 4B).

### MetaboDiff results

**Figure 6:**
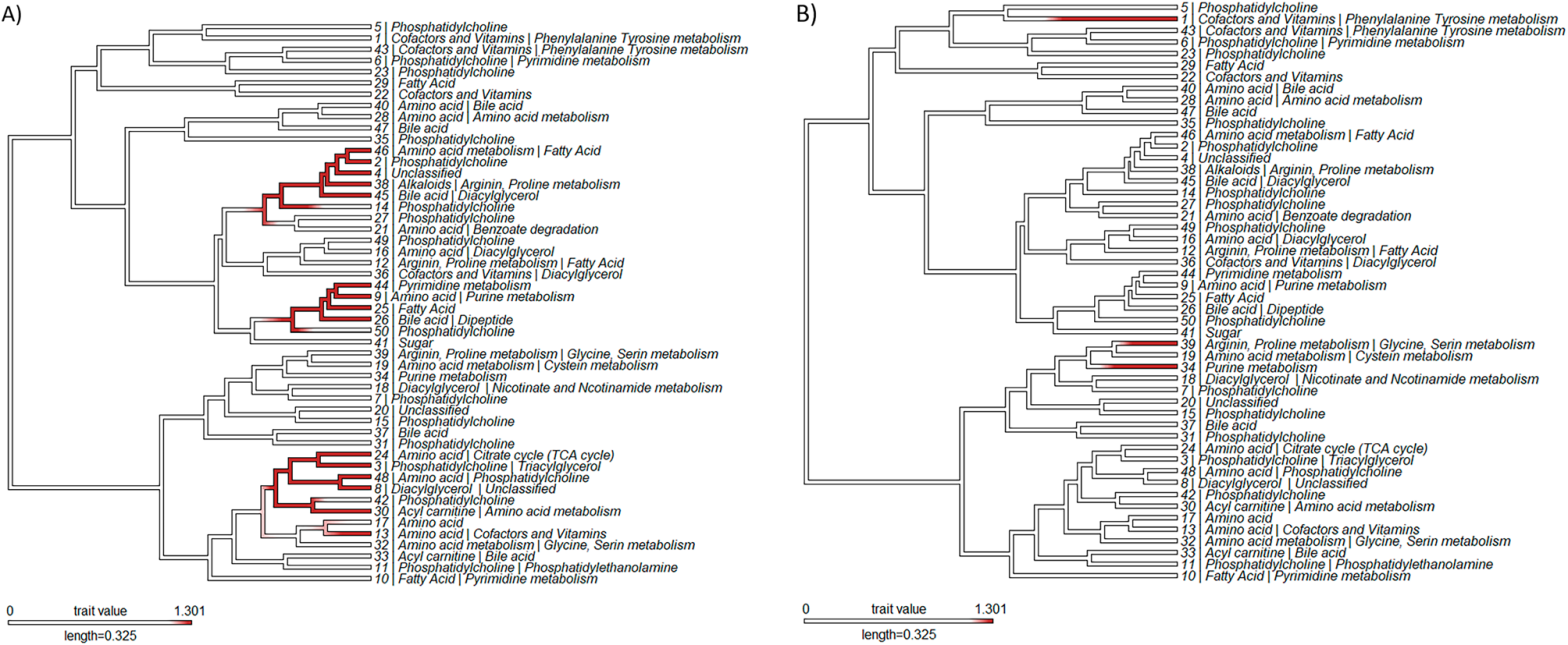
MetaboDiff clustering analysis. Metabolic correlation modules associated with **(A**) GAN diet versus CRF1 diet, and (**B**) with or without administration of GH.

Differential metabolomics analysis was performed using MetaboDiff version 0.9.3 using default parameters^24^. Imputation and variance-stabilizing normalization were performed, and metabolites were annotated by querying against the small molecular pathway database (SPMDB) using HMDB, KEGG, and ChEBI identifiers. Metabolic correlation modules associated with metabolism of amino acids, pyrimidines, and fatty acids were identified corresponding to GAN diet (Fig. 6A, Suppl. Fig. 5A), while a small number of metabolic correlation modules associated with amino acid metabolism were identified with respect to GH treatment (Fig. 6B, Suppl. Fig. 5B).

#### Multi-omics analysis: mixOmics results

**Figure 7:**
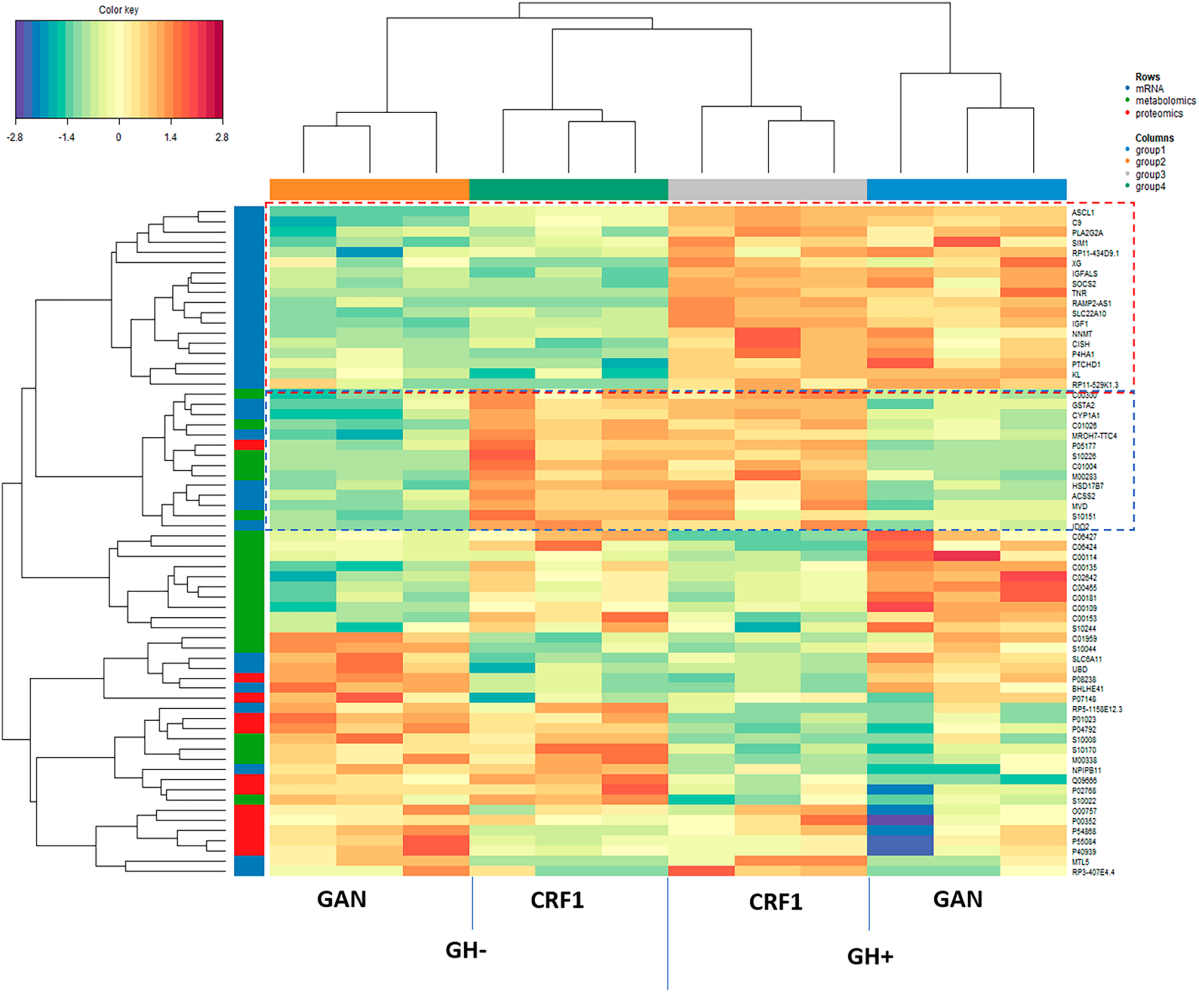
Multi-omics analysis using mixOmics. DIABLO heatmap showing relationships among gene, protein, and metabolite profiles with respect to growth hormone and GAN diet. The box outlined in red represents features associated with GH, while the box outlined in blue represents features associated with diet.

Multi-omics analysis of gene expression, proteomics, and metabolomics data was performed using mixOmics version 6.18.1^25^. DIABLO is a supervised method based on multiblock sparse partial least squares discriminant analysis that accounts for the heterogeneity among omics-specific datasets in evaluating the association with a categorical outcome variable. Heatmap results revealed clustering by diet and GH (Fig. 7). GSEA analysis of genes associated with GH indicated enrichment of the Hallmark IL2/STAT5 signaling pathway (box 1), while genes associated with GAN diet (box 2) were enriched for Hallmark cholesterol synthesis and fatty acid metabolism pathways.

#### Multi-omics analysis: MiBiOmics results

**Figure 8:**
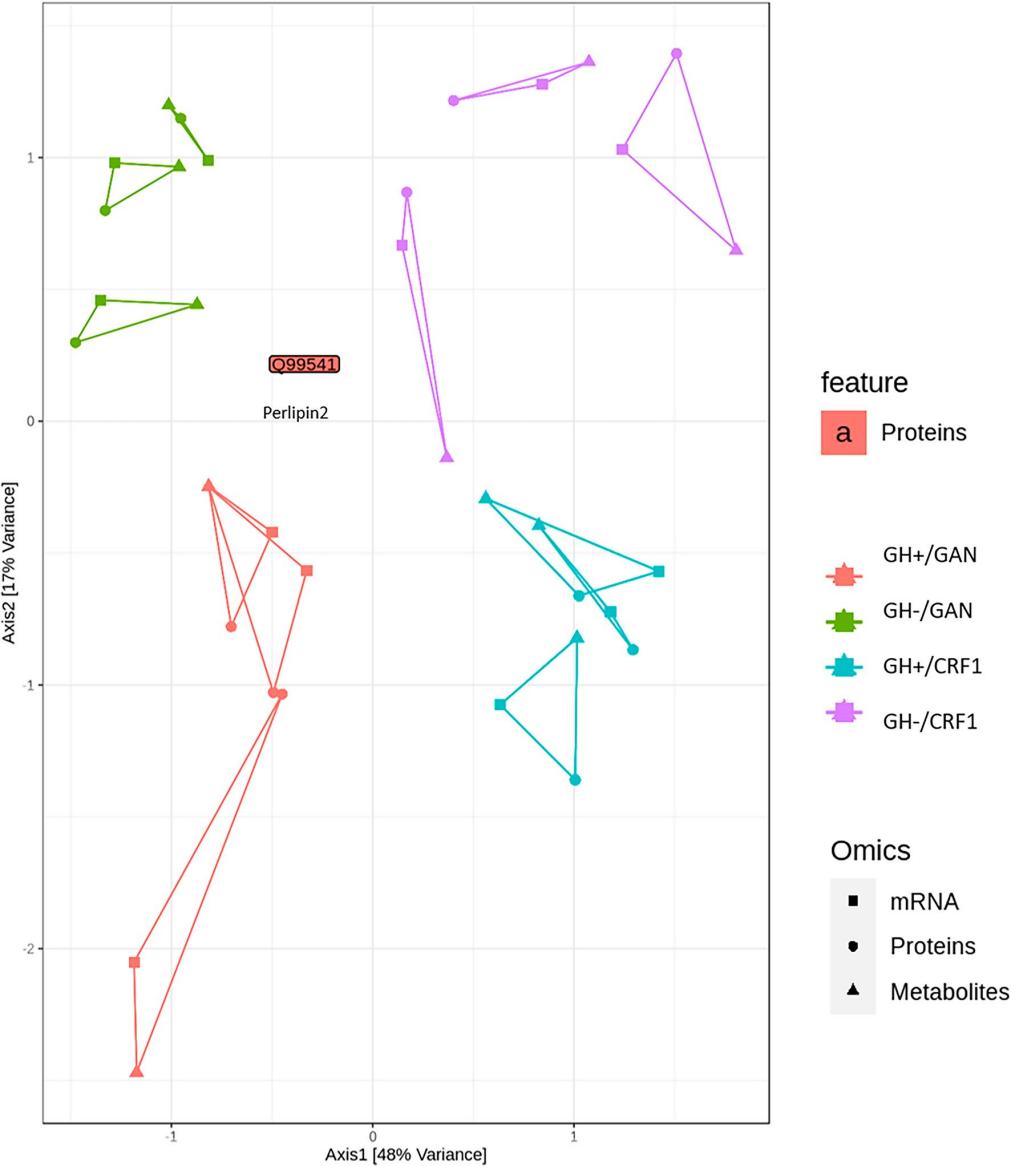
Multi-omics analysis using MiBiOmics. Each triangle represents a different mouse, with each vertex representing a different omics type. Perlipin-2 was identified as a co-inertia driver.

MiBiOmics generalizes WGCNA to other types of omics data and provides a method for joint multi-omics analysis of up to three data sets^26^. The three-way omics coordinates for each mouse clustered together with respect to treatment group with no overlap among groups, although the mRNA and metabolomics coordinates diverged from the proteomics coordinate for one of the GH+/GAN mice (Fig. 8). MiBiOmics analysis identified perilipin-2 (encoded by PLIN2) as a co-inertia driver (Fig. 8).

## Discussion

In this study, we attempted to develop a mouse model of fatty liver using an existing human hepatocyte chimeric mouse model that we have previously used successfully to investigate hepatitis B and C virus infection and evaluate antiviral drugs. The tendency of human hepatocyte chimeric mice to exhibit mild to moderate fatty liver due to the lack of human GH and the inability of transplanted human hepatocytes to respond to mouse GH makes it possible to establish a range of fatty liver conditions. When mice were supplemented with human GH, fatty liver conditions tended to be milder, whereas GH− mice fed that were fed a high-fat GAN diet exhibited a more severe form of fatty liver. Abdominal echocardiography revealed a significantly lower liver/kidney ratio in mice that were either fed a CRF1 diet or that were treated with human GH, although addition of GH in GAN-fed mice did not significantly reduce the liver/kidney ratio. This suggests that GH is necessary to suppress accumulation of fat in hepatocytes but is not sufficient to prevent excess fat accumulation when fed a high-fat diet.

Histological inspection and electron microscopy revealed corresponding changes in cell and tissue morphology. Sirius Red staining revealed fibrosis radiating from the venule and surrounding individual hepatocytes in GH−/GAN mice, as well as to a lesser extent in GH−/CRF1 mice and GH+/GAN mice, but not in GH+/CRF1 mice. Importantly, this indicates that fat accumulation within transplanted hepatocytes is accompanied by corresponding changes in the extracellular matrix consistent with mild fibrosis, which is expected to result in deterioration of liver function and suggests that this model can successfully recapitulate several essential aspects of the pathophysiology of fatty liver within an experimental timeframe.

Electron microscopy showed similar findings, with large, rounded hepatocytes with displaced nuclei in a GH−/GAN mouse compared to a GH+/CRF1 mouse. Mitochondria in the GH−/GAN mouse appeared to be less numerous and tended to be either smaller or occasionally much larger than in the GH+/CRF1, with less conspicuous adjacent endoplasmic reticulum. The presence of elongated or enlarged mitochondria called megamitochondria with crystalline inclusions is characteristic of NAFLD and may be due to fusion of mitochondria or incomplete fission indicative of mitochondrial dysfunction^27,28^. Electron microscopy also showed corresponding changes in the pericellular space, with the space between cells appearing wider and more electron-dense with the appearance of stunted microvilli.

Results from this study are generally consistent with previous reports and known findings, suggesting that the model provides a reasonable representation of several aspects of human fatty liver. The frequency of NAFLD is known to increase in adults with GH deficiency^29^, and GH replacement therapy has been shown to reduce NASH conditions^30^. GH-deficient rats are also known to develop NASH, which improves with administration of GH^31^. Similarly, NASH symptoms have been shown to improve with administration of IGF-1, the primary downstream target of GH in the liver^31,32^.

The advantage of this model is that various degrees of fatty liver can be induced depending on the type of diet and the use of GH supplementation. Liver tissue can be analyzed with a range of omics methods to elucidate pathological conditions and examine drug efficacy. The effect of a candidate drug on the degree of fatty liver could also be evaluated in vivo using ultrasound.

These qualities facilitate use of the animal model to investigate mechanisms leading to MAFLD development. Histological findings indicated macrovesicular steatosis, which likely reflects increased synthesis of triglycerides due to accumulation and/or reduced oxidation of free fatty acids^33^. These conditions are reflected as changes in patterns of gene expression and protein and metabolite profiles among mice in different treatment groups.

RNA-Seq results revealed modest changes in gene expression following addition of GH in mice. However, robust changes in gene expression were observed due to diet in GAN-fed versus CRF1-fed mice. Importantly, in GAN-fed mice, the genes that were most strongly up-regulated differed greatly depending on whether or not GH was administered. For example, tyrosine hydroxylase (TH), which is involved in the synthesis of catecholamines involved in upstream regulation of GH^34^, was up-regulated 64-fold in GH-treated mice but did not differ significantly in untreated mice. Conversely, acyl CoA synthetase-1 (ASCL1), which is involved in uptake of long-chain fatty acids^35^, was up-regulated 62-fold in untreated mice versus 11-fold in GH-treated mice.

While RNA-Seq focuses on the most strongly differentially expressed genes, smaller coordinated changes in gene expression may yield greater insight into the overall metabolic state. WGCNA identified distinct modules corresponding to the effect of GH and GAN diet, represented by eigengenes IGF-1 and SEMA7A, respectively. GH secreted from the pituitary gland induces expression of IGF-1 in hepatocytes through JAK2/STAT5 signaling via the GH receptor. Activation of the IGF-1 receptor induces lipogenesis and glycogen synthesis and suppresses lipolysis and gluconeogenesis. Lower IGF-1 expression has been found to be associated with increasing NAFLD severity^36^. SEMA7A has been shown to be protective against high-fat diet-induced obesity, while SEMA7A deletion resulted in increased steatosis and insulin resistance in a mouse model^37^. Furthermore, SEMA7A R148W polymorphisms induce lipid droplet accumulation in mice and were found to be associated with NAFLD in patients through enhanced uptake and synthesis of fatty acids and triglycerides^38^.

Proteomics results indicate that the level of HIG1 domain family member 2A (HIGD2A) protein was 21-fold down-regulated in GAN-fed mice compared to CRF1-fed mice. This hypoxia-associated inner mitochondrial membrane protein has been suggested to be protective against lipotoxicity following exposure to a high-fat diet^39^. Conversely, the level of transmembrane member 16A (TMEM164) protein was 12-fold higher in GAN-fed mice. TMEM164 has been shown to promote steatosis via regulation of oxidative stress through calcium-activated chlorine channels and is up-regulated in patients and mice with NAFLD^40^.

Comparison of metabolite profiles revealed several trends. Compared to CRF1-fed mice, untreated GAN-fed mice showed a sevenfold increase in TG(56:4) triacylglycerol. Conversely, GAN-fed mice treated with GH showed a 14-fold increase in PC(40:6) and other phosphatidylcholines. Essential phospholipids rich in phosphatidylcholine are associated with reduced steatosis and are sometimes used as a treatment for NAFLD/MAFLD^41^. GH administration in CRF1-fed mice was associated with a fivefold change in 1-methylnicotinamide (MNA), which promotes fatty acid beta-oxidation and decreases lipid levels in the liver by stabilizing sirtuin 1^42^. Suppression of MNA metabolism has been suggested as a means to help reverse fatty liver^43^. GH administration in GAN-fed mice was associated with a sixfold change in ethylmalonic acid. High levels of ethylmalonic acid are associated with short chain acyl-CoA dehydrogenase deficiency, a disorder of fatty acid metabolism. MetaboAnalyst and MetaboDiff results indicate enrichment of metabolite sets associated with amino acid metabolism and the urea cycle in GH + versus GH− mice, revealing differences in protein metabolism. When food availability is limited, GH and IGF-1 promote protein retention by inducing preferential use of lipids as an energy source^44^.

While separate analysis of individual omics data sets is essential, joint analysis provides unique insight and helps to reveal trends that “echo” across two or more omics data sets. Integrating different types of omics data poses statistical and combinatorial challenges. MiBiOmics addresses this problem by identifying modules within each data set and then focusing on the correlations among modules. Using this approach, MiBiOmics identified perilipin-2 as a co-inertia driver. This protein assists in formation of lipid droplets and contributes to fatty liver by promoting uptake of fatty acids^45^, suggesting that it represents a potential target for development of MAFLD treatments. Perilipin-2 has been found to play an important role in initiation and progression of NAFLD/MAFLD^46^, while deletion of perilipin-2 in a mouse model reduced triglyceride and cholesterol levels in the liver by suppressing genes involved in lipogenesis and cholesterol biosynthesis^47^.

WGCNA-based methods such as MiBiOmics provide an unsupervised method to combine different types of omics data using a network-based topology, whereas table-based methods such as mixOmics DIABLO provide a supervised method to combine multiple omics data sets while accounting for statistical differences among omics data types. The DIABLO heatmap reveals the joint contributions of each omics data set and shows that mRNA, protein, and metabolite profiles vary in a coordinated way with respect to diet and GH. The mice are clustered first by diet and then by GH, suggesting that administration of GH does not fully relieve the effects of a high-fat diet but does favorably influence metabolic and gene expression profiles in the liver.

Taken together, three complementary high-throughput methods identified a number of molecules known to be associated with lipotoxicity and NAFLD/MAFLD and revealed several potential new targets. Multi-omics analysis, histological findings, and ultrasound imaging reflected coordinated changed in gene expression, protein, and metabolite profiles associated with fatty liver in a mouse model.

### Limitations of the study

While we feel that this animal model provides a useful system to investigate MAFLD in vivo, we acknowledge that this study suffers from several important limitations. The lack of a standard murine model as a control and the limited mechanistic insight that can be gained from high-throughput methods limits the direct translational benefits of the study, but we have reported changes in specific genes, proteins, and metabolites of potential interest that provide a basis for confirmation in future mechanistic studies. We had also anticipated a more robust effect in response to administration of human GH, but the effect of GH tended to be overshadowed by the larger effects of the GAN diet, and the small sample size limited the power of the study to assess the effects of GH in detail. Generally, the samples clustered strongly with respect to treatment group, but mRNA and metabolite profiles varied to a greater extent in of one of the GH+/GAN mice compared to other mice in the same group (Fig. 8, lower left). This might be related to variation in human hepatocyte repopulation rates (Suppl. Fig. 2), reflecting the need for additional replicates and further large-scale studies. Finally, we neglected to include longitudinal serum measurements of metabolic parameters associated with MAFLD or GH treatment (e.g., insulin, glucose, fatty acids, and triglycerides), which might have yielded greater insight into the joint multi-omics effects of diet and administration of GH, although we argue that the capacity to be able to perform experiments of this kind represents one of the potential benefits of this model and should be pursued in future studies.

## Conclusions

This study introduces a human hepatocyte chimeric mouse model of fatty liver that can be used to investigate the regulation and pathogenesis of fat accumulation in the liver. The results from separate and combined transcriptomics/proteomics/metabolomics analysis revealed distinct gene expression and protein/lipid profiles associated with each treatment group. The effect of GAN diet was greater overall than the effect of supplementation with GH; however, addition of GH reduced fatty liver conditions, suggesting that this model may be useful for evaluating the effect of investigational compounds on resolution of fatty liver.

## Materials and methods

### Preparation of chimeric mice

Human hepatocyte chimeric mice were prepared as described previously^48^. Briefly, human hepatocytes were transplanted into cDNA-uPA +/ +/SCID +/ + (uPA/SCID) mice, achieving a re-population rate of ≥ 70%. Human serum albumin concentrations were monitored by serial mouse blood collection (Suppl. Fig. 2). One of the mice had a slightly lower human albumin rate that decreased further over time. All mice used in the study were male. Mice were humanely euthanized at 8 weeks using isoflurane.

### Statement of ethical approval

All animal protocols described in this study were performed in accordance with the ARRIVE Guidelines, the Guide for the Care and Use of Laboratory Animals (https://grants.nih.gov/grants/olaw/guide-for-the-care-and-use-of-laboratory-animals.pdf), and the local committee for animal experiments. The experimental protocol was approved by the Ethics Review Committee for Animal Experimentation of the Graduate School of Biomedical Sciences, Hiroshima University (A14-195).

### Administration of a high-fat diet

Mice in the normal diet group were fed CRF1 diet (gamma ray irradiated CRF1, Oriental Yeast co., Ltd) orally ad libitum. Mice in the high-fat diet group were fed Gubra amylin NASH (GAN) diet, an obesogenic trans-fat rodent diet in which the caloric content consists of 40% fat (mainly palm oil), 20% fructose, and 2% cholesterol. Palm oil is a vegetable oil characterized by a high content of saturated fatty acids, particularly palmitic acid.

### Administration of human growth hormone

Human growth hormone was administered to three mice in the GAN and CRF1 diet groups using an ALZET syringe pump (ALZET International Distributors, Tokyo, Japan) implanted subcutaneously.

### Abdominal echocardiography

Assessment of fatty liver in SCID mice with transplanted human hepatocytes is difficult due to the presence of numerous fat droplets. The degree of fatty liver was estimated using abdominal echocardiography using a SonoScope S6V (Shoei Japan, Co., Tokyo, Japan). Ultrasound images were analyzed using ImageJ version 153 to calculate the liver : kidney intensity ratio (Fig. 1A). Statistical analysis was performed using R version 4.2.3.

### Histology with Sirius red staining

Liver samples from mice fed a CRF1 diet versus GAN diet and treated with and without GH were stained with hematoxylin and eosin and then stained with Sirius red (Sigma-Aldrich, Tokyo, Japan) following the manufacturer’s instructions. Sirius red is an azo dye that stains red in the presence of collagens I and II and green in the presence of collagen III.

### Electron microscopy

Electron microscopy was performed by Tokai Electron Microscopy, Inc. Liver samples from a CRF1 diet/GH+ mouse and a GAN diet/GH−  mouse were sliced into 70 nm sections using an ultramicrotome with a diamond knife (Ultracut UCT; Leica, Vienna, Austria). Sections were mounted on copper grids and stained with 2% uranyl acetate for 15 min and then washed with distilled water. Then the sections were stained with Lead stain solution (Sigma-Aldrich Co., Tokyo, Japan) for 3 min. Sections were photographed by CCD camera (EM-14830RUBY2; JEOL Ltd., Tokyo, Japan) using a transmission electron microscope (JEM-1400Plus; JEOL Ltd., Tokyo, Japan) using a 100 kV acceleration voltage.

### Multi-omics analysis

After sacrifice, liver tissue containing human hepatocytes was explanted from chimeric mice for multi-omics analysis in order to detect coordinated changes in gene, protein, and metabolic profiles. For each mouse, proteomic and metabolomic analyses were conducted on adjacent samples of the same liver tissue used for transcriptomic analysis.

### RNA-sequencing

RNA from humanized mouse liver tissue was extracted and purified by TRIZOL or TRIZOL-LS (ThermoFisher/Invitrogen, 15,596,026 or 10,296,028). Quality control of RNA was performed using Nanodrop and Agilent Bioanalyzer RNA 6000 Pico Kit (5067-1513). Next, 500 ng total RNA was used for rRNA-depletion (NEB, E6310), followed by directional library synthesis (NEB, E7420). This library was quality controlled using Agilent Bioanalyzer, DNA High-sensitivity kit (5067-4626). RNA-sequencing was performed by Illumina NextSeq500 with a standard of High-output kit v2, 2 × 36. Raw data were exported as 8 compressed FASTQ files per sample (4 imaging section × Read 1 + 2). Data analysis was performed using CLC Genomics Workbench (v10.1.1). FASTQ files were concatenated and imported as paired-end reads. Reads were mapped to the human hg19 reference sequence, which contains annotations for 57,773 genes and 173,446 transcripts. EdgeR version 3.41 was used to analyze pairwise differential gene expression. False discovery rate (FDR) < 0.05 was considered statistically significant. Gene enrichment analysis (Gene Ontology and pathway analysis) was performed to investigate the biological function of differentially expressed genes.

### Proteomics

DIA proteomic analysis was performed by Kazusa Genome Technologies, Inc. In brief, the preprocessing stage involved protein extraction and peptide fragmentation by trypsin. Subsequently, MS measurements were performed by nano liquid chromatography with tandem MS (UltiMate 3000 RSLC-nano LC System, Q Exactive HF-X, Thermo Fisher Scientific Inc.). From the acquired MS data, peptides and proteins with both peptide and protein false discovery rate (FDR) below 1% were identified and quantified using Scaffold DIA (Proteome Software, Inc., Portland, OR, USA). Protein quantification data obtained via DIA proteomic analysis were analyzed and plotted using Microsoft Excel. Gene ontology (GO) enrichment analysis was performed using RDAVIDWebService version 1.0.0.

### Metabolomics

Metabolomics analysis was performed by LSI Medience (Kamisu, Ibaraki, Japan). LC–MS was done using a LC system (HP1200; Agilent Technologies) equipped with a C18 column (2 µm, 50 mm × 2.0 mm ID, CAPCELL PAK C18 IF; Shiseido, Tokyo, Japan) coupled with an electrospray ionization quadrupole time-of-flight mass spectrometer (6520; Agilent Technologies). Solvent A was composed of 5 mM ammonium acetate aqueous solution, whereas solvent B was acetonitrile. Metabolites were eluted at a flow rate of 0.2 mL/min at 40 °C with a linear gradient of 10–90% solvent B over 10 min, followed by a further 5-min hold at 100% solvent B. The mass spectrometer was operated in positive and negative scan modes (*m/z* 60 to 1200) with a capillary voltage of 3500 V. The nebulizing gas pressure was 40 psi and the dry gas flow rate was 8 L/min at 350 °C. Ionic metabolites were measured in the positive mode of a CE-time-of-flight mass spectrometer (6520; Agilent Technologies). Metabolites were separated in a fused-silica capillary (50 µm i.d. × 100 cm total length; GL Science, Tokyo, Japan) filled with 1 mol/L of formic acid aqueous solution (cation mode), or 20 mM ammonium formate and 20 mM ammonium acetate aqueous solution (pH 10, anion mode) as the electrolyte. The sample solution was injected at 5 kPa for 15 s (~ 15 nL), and a voltage of 30 kV was applied. The capillary tray and sample tray were maintained at room temperature and at 5 °C, respectively. The sheath liquid was methanol/water (50% *v/v*) containing 5 mM ammonium acetate. The CE-time of flight mass spectrometer was operated in positive and negative scan modes (*m/z* 60–1200). The capillary voltage was set at 3500 V, and the nitrogen gas (heater temperature = 250 °C) flow rate was set at 10 L/min. The MS data file was converted to CSV format with csv convertor (Agilent Technologies). All peak positions (retention time and *m/z*) and areas were calculated by Markeranalysis (LSI Medience). All peak areas were aligned into one data sheet, and the errors of peak intensities were corrected by internal standards. Noise peaks were deleted by comparison with the peaks detected in blank samples. Metabolites were identified by comparing the retention time and *m/z* with the standard data set established by LSI Medience. Mean fold-change and the Student’s *t*-test were carried out for all detected peaks.

### Supplementary Information

Supplementary Tables.

Supplementary Figures.

## Data Availability

All data are available upon request. RNA-Seq FASTQ data have been uploaded to the Sequence Read Archive (accession PRJNA972864).

## Abbreviations

SCID: Severe combined immunodeficiency
uPA: Urokinase-type plasminogen activator
MAFLD: Metabolic-associated fatty liver disease
NAFLD: Non-alcoholic fatty liver disease
NASH: Non-alcoholic steatohepatitis
