# Supplementary material for: Multi-omics analysis of a fatty liver model using human hepatocyte chimeric mice
Source: Sci Rep. 2024 Feb 9;14:3362. doi: 10.1038/s41598-024-53890-8 (PMC10858249; doi:10.1038/s41598-024-53890-8)
Supplement: Supplementary file 2 — Supplementary Figures. [file 41598_2024_53890_MOESM2_ESM.docx]

**Suppl. Fig. 1. Change in body mass of human hepatocyte chimeric mice.** Group 1 and 2 mice were fed a high-fat GAN diet while group 3 and 4 mice were fed a normal CRF1 diet. Groups 1 and 3 were administered human growth hormone while groups 2 and 4 were not.

**Suppl. Fig. 2. Change in human serum albumin levels in human hepatocyte chimeric mice.** Group 1 and 2 mice were fed a high-fat GAN diet while group 3 and 4 mice were fed a normal CRF1 diet. Groups 1 and 3 were administered human growth hormone while groups 2 and 4 were not.

**Suppl. Fig. 3. Abdominal echocardiography of liver/kidney ratio with respect to GAN vs CRF1 diet and GH treatment in human hepatocyte chimeric mice.**


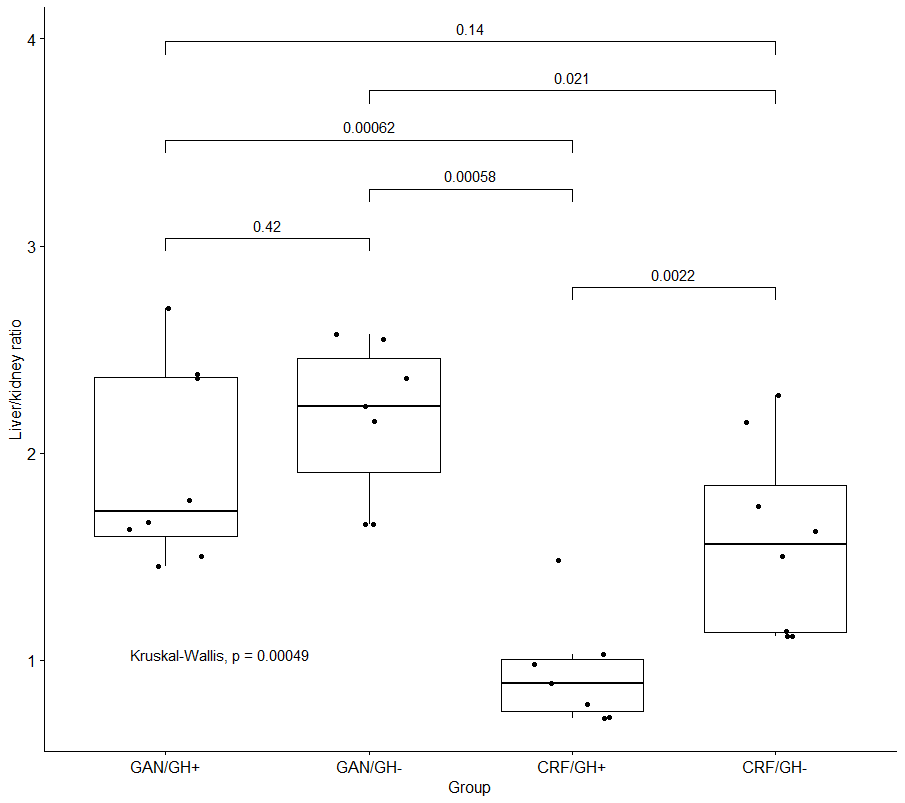


**Suppl. Fig. 4. Individual metabolites associated with GAN diet and GH using the MetaboAnalyst statistical analysis with metadata module.** A) Metabolites associated with GAN diet. B) Metabolites associated with administration of GH.


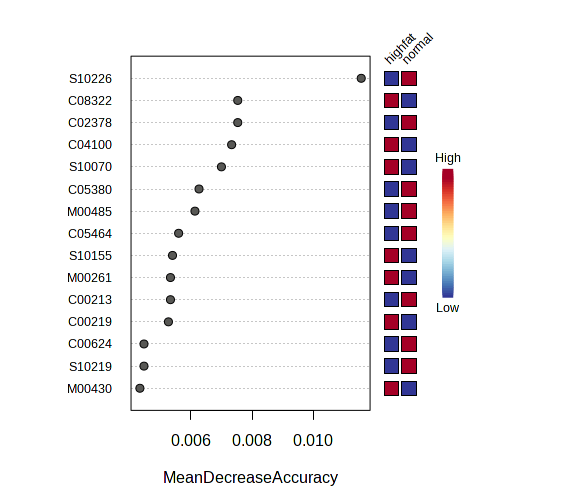

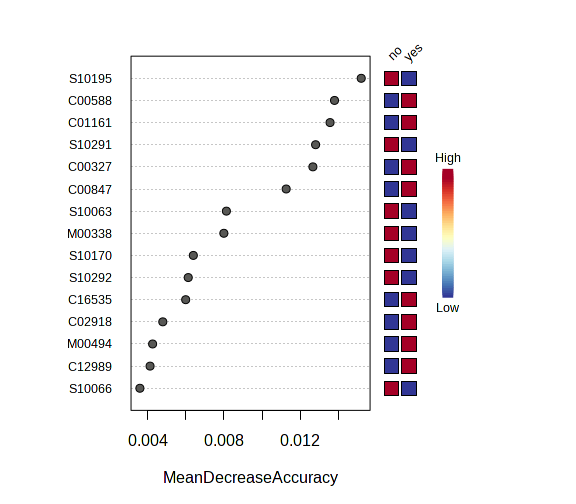


A)

B)

**Suppl. Fig. 5. MetaboDiff volcano plots.** A) Metabolites associated with GAN diet. B) Metabolites associated with GH administration.


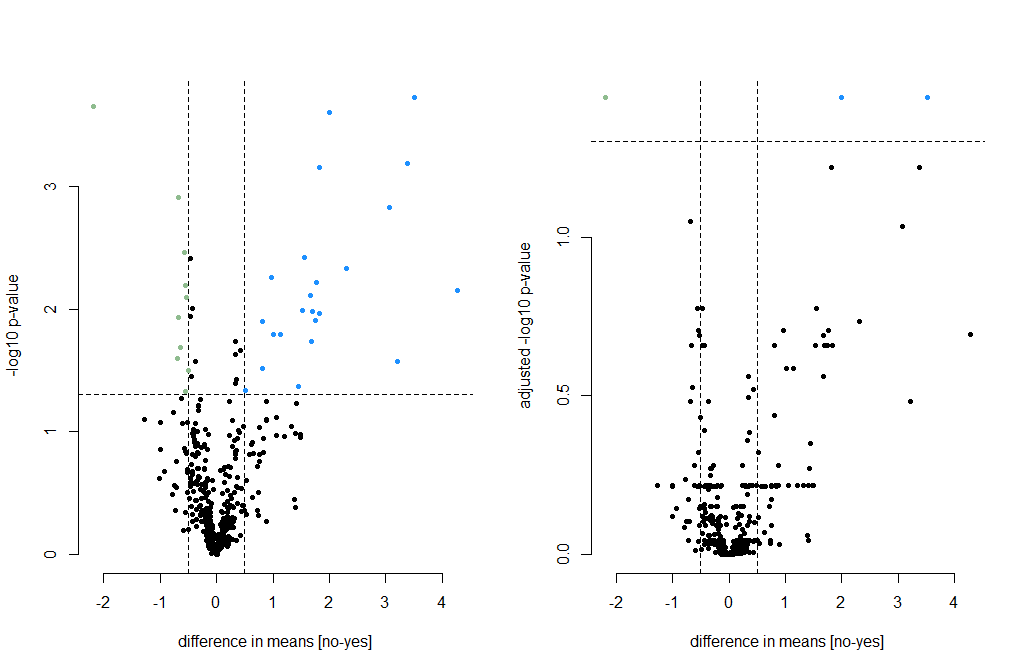


A)

**GAN diet**


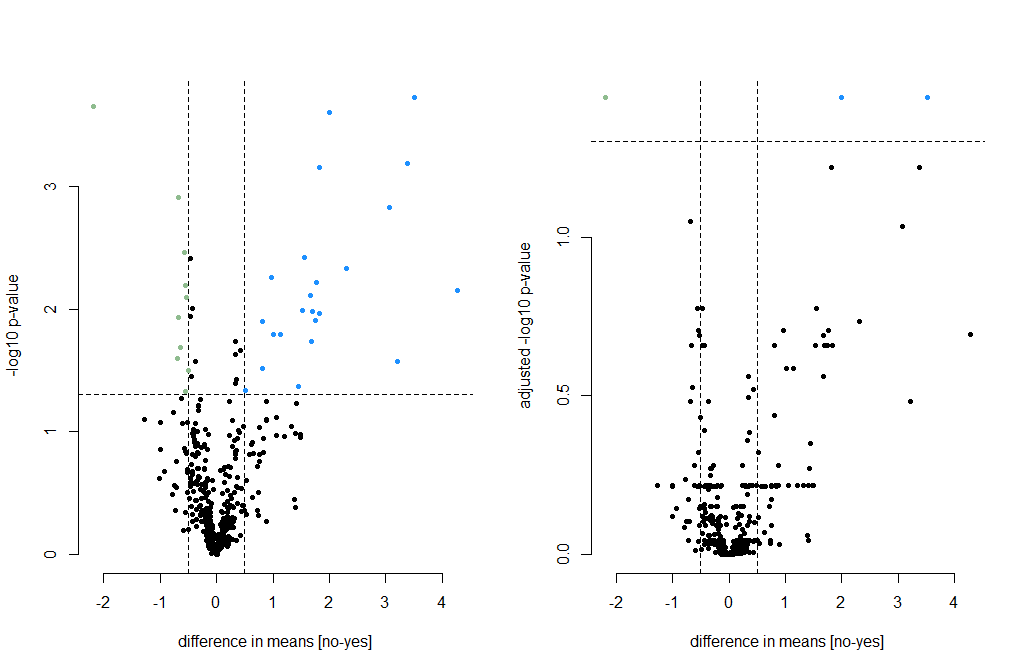


**Growth hormone**

B)
